# IgG In Saliva, GCF, and Serum in Young Patients With Grade C Molar Incisor Pattern Periodontitis

**DOI:** 10.1002/cre2.70117

**Published:** 2025-03-30

**Authors:** Meaad M. Alamri, Gordon Proctor, Luigi Nibali

**Affiliations:** ^1^ Periodontology Unit, Centre for Host Microbiome Interactions, Faculty of Dentistry, Oral & Craniofacial Sciences King's College London London UK; ^2^ Dental Health Department, College of Applied Medical Sciences King Saud University Riyadh KSA

**Keywords:** GCF, grade C, immunoglobulin, molar‐incisor pattern, periodontitis, saliva

## Abstract

**Objective:**

This cross‐sectional study aimed to investigate immunoglobulin G levels in saliva, gingival crevicular fluid, and serum samples from young patients with grade C molar incisor pattern periodontitis (C/MIP) and age‐matched periodontitis‐free controls.

**Methods:**

Saliva, gingival crevicular fluid, and blood samples were collected from 62 patients, divided into 31 cases and 31 periodontitis‐free age‐matched controls. Saliva and blood samples were centrifuged to extract supernatant and serum. Gingival crevicular fluid periopapers were eluted. Human total immunoglobulin G levels were assessed using an Enzyme‐Linked Immunosorbent Assay.

**Results:**

After adjusting for covariates, cases had higher Immunoglobulin G levels in saliva (*p* = 0.005), gingival crevicular fluid (*p* < 0.001) than controls; however, serum did not reach the significant threshold (*p* = 0.137). Among other factors contributing to immunoglobulin G levels, males had higher serum immunoglobulin G than females (*p* = 0.018), and serum immunoglobulin G levels increased with age (*p* = 0.033). Gender and ethnicity subgroup analyses revealed that C/MIP males had higher saliva IgG (*p* = 0.018) than control males, and both genders had higher GCF IgG than controls (*p* ≤ 0.001). C/MIP Caucasians had elevated saliva (*p* = 0.011) and GCF IgG p = (0.003) compared to the controls, and Asians had higher GCF IgG than the controls (*p* = 0.011).

**Conclusion:**

This study shows for the first time that C/MIP cases have higher Immunoglobulin G levels than controls in saliva and gingival crevicular fluid, confirming its association with C/MIP pathogenesis and suggesting that it could be a potential biomarker in grade C molar incisor pattern periodontitis. Further research on a larger sample size is needed.

## 1 Introduction

Grade C molar incisor pattern periodontitis (C/MIP) is an advanced form of periodontal disease according to the 2017 classification of periodontal and peri‐implant diseases and conditions (Papapanou et al. 2018). It is a severe inflammatory disease characterized by rapid attachment loss, deep periodontal pockets, and bone resorption affecting molars and incisors, causing tooth mobility and eventually tooth loss if left untreated (Papapanou et al. 2018). Periodontal diseases are prevalent in adults, unlike in children (Albandar and Tinoco 2002; Baiju et al. 2019; Raitapuro‐Murray et al. 2014). C/MIP affects children with primary dentition and can progress to permanent dentition, increasing the risk of premature tooth loss at a young age (Muppa et al. 2016; Reis et al. 2021).

C/MIP pathogenesis is not entirely clear, as factors beyond simple plaque accumulation play an important role (Albandar 2014; Fine et al. 2018). A combination of factors, including genetics, immune response, and pathogenic microbes, is believed to trigger C/MIP development (Albandar 2014; Kinane et al. 1999; Lertpimonchai et al. 2017). The immune response is the core system for defending the host against pathogens (Chaplin 2010). IgG is an essential antibody in the adaptive immune system (Chaplin 2010). It is a glycoprotein that can easily travel from the blood to different tissues to neutralize bacteria by binding to them, marking them for phagocytoses and thus preventing them from causing infections (Justiz Vaillant et al. 2024; Marshall et al. 2018). As the name indicates, C/MIP is localized to molars and incisors; however, when IgG is no longer capable of eliminating bacteria such as *Aggregatibacter actinomycetemcomitans*, the disease may extend to become generalized (Fine et al. 2018; Gunsolley et al. 1987). Therefore, the excessive B‐cell transformation into plasma cells increases IgG production, which indicates an ongoing inflammation (Subbarao et al. 2019; Takahashi et al. 1997).

When comparing young C/MIP with older C/MIP and chronic periodontitis, serum total IgG was statistically significantly higher in a group of older patients with aggressive periodontitis than chronic periodontitis (Dhoondia et al. 2018) and apical periodontitis than controls (Matos‐Sousa et al. 2024). Moreover, serum IgG to *Porphyromonas gingivalis* was also suggested as a diagnostic biomarker for chronic and aggressive periodontitis (Kudo et al. 2012; Stathopoulou et al. 2015). However, the majority of studies on IgG in periodontitis focused on either IgG variations to specific pathogens or IgG subclasses (IgG_1_–IgG_4_), while few studies investigated total IgG. This highlights the lack of data on total IgG assessment.

Our previous work systematically searched the literature to identify diagnostic biomarkers for C/MIP (Alamri et al. 2023). Only two studies were eligible for meta‐analysis with low heterogeneity, and they showed a significant elevation of serum IgG in C/MIP compared to the controls (Alamri et al. 2023). Very few studies investigated total IgG levels in young patients ( ≤ 25 years old) with C/MIP in serum and GCF but not in saliva (Alamri et al. 2023). These studies were conducted over two decades ago, and none of them investigated all three of these bodily fluids. This conclusion highlighted the gap in the literature and the need to assess IgG in other body fluids (Alamri et al. 2023). In the era of personalized medicine, investigating IgG levels in young populations could give insights into the pathogenesis of C/MIP and could potentially be used for future diagnostic tests. Therefore, this study aimed to assess IgG levels in three samples, including saliva, GCF, and serum samples, collected from young C/MIP cases and age‐matched controls. Given the IgG role in immunity, we hypothesized that IgG concentrations would be significantly higher in C/MIP than in controls.

## 2 Materials and Methods

### 2.1 Ethical Approval

This study participants were recruited through the Oral, Dental and Craniofacial Biobank at King's College London in London. The Biobank is ethically approved by the East of England‐Cambridge East Research Ethics Committee (reference 20/EE/0241). Specific approval for the current cross‐sectional study was obtained from the biobank management committee (Biobank reference REF018).

### 2.2 Patient Recruitment

Young patients seen in the dental clinics at Guy's Hospital in London were invited to participate in the Biobank. Biobank patients were included in this study if they fulfilled the following eligibility criteria: age ≤ 25 years, any ethnicity (e.g., Caucasian, Asian, Afro‐Caribbean, and Mixed), never smoked, did not have a medical condition associated with periodontitis (e.g., diabetes, rheumatoid arthritis, cardiovascular diseases) not under medications related to periodontitis (e.g., calcium channel blockers, corticosteroids, anticoagulants), and absence of pregnancy and lactating. Potential patients were given detailed information sheet about the study, followed by a consent form. Patients' consent was required before samples and data collection. C/MIP and controls had the same eligibility criteria except for the periodontal diagnosis, as C/MIP had to be clinically diagnosed with C/MIP and controls to be clinically free from periodontitis.

### 2.3 Clinical Examination

Patients' oral examination was carried out at Guy's Hospital. A complete medical and dental history was recorded, HbA1C, height, weight, and waist measurements were taken to confirm the absence of diabetes and calculate the body mass index. Additionally, radiographs were taken according to clinical needs, in addition to the recording of the following clinical measurements using a UNC‐15 periodontal probe: periodontal probing depth (PPD) measured from gingival margin to the base of sulcus/pocket, recession (REC) measured from the CEJ to the gingival margin, clinical attachment loss (CAL) calculated by adding PPD to REC, basic periodontal examination (BPE), bleeding on probing (BOP), basic erosive wear examination (BEWE), presence of mobility, furcation involvement, implants or dentures. Radiographs and clinical measurements were used to confirm the diagnosis.

### 2.4 Definitions of Study Groups

**C/MIP:** Stages II–IV were included in this group if they have ≥ 2 nonadjacent sites with CAL ≥ 3 mm, ≥ 15% RBL, and PPD ≥ 5 mm with rapid progression of the periodontitis measured by having > 1.0 when dividing the percentage of bone loss over the age, and the periodontal destruction exceeding the present plaque accumulation (Papapanou et al. 2018). MIP pattern should not affect more than two teeth other than 1st molars and incisors. In a few cases, we have assumed MIP based on the observed disease pattern, even if more than two teeth—excluding first molars and incisors—were affected. This was done to ensure consistency in classification while considering the overall clinical presentation.

**Controls:** BOP of any percentage but with PPD ≤ 3 mm and no evidence of CAL or RBL (Caton et al. 2018).

### 2.5 Sample Collection and Processing

**Saliva samples**: Samples were collected before the oral examination. Patients refrained from eating or drinking at least 1 h before sample collection. Five milliliters of unstimulated saliva was collected using a passive drooling method. Each patient was asked to rinse with water before allowing saliva to pool in the mouth and drooling it into a 20 mL tube for 10 min. Patients who provided 5 mL before the end of the 10 min were stopped at that point. The sample was aliquoted into four Eppendorf tubes of 1 mL each and stored at −80°C.

**GCF samples**: For each patient, the mesiobuccal surface of all first molars (diseased sites for C/MIP and healthy sites for controls) was sampled by isolating the teeth with a cotton roll, drying them, and then inserting a periopaper into the pocket/sulcus for 30 s. The volume of the absorbed crevicular fluid in each periopaper was measured using a periotron. All four periopapers were stored in one Eppendorf tube at −80°C for later elution.

GCF volume (V_GCF_) of the periostrips was calculated using the equation *y* = *a* + *bx* ^*c*^, in which *y* is the periotron reading in units, *a* is 0 for the intercept, *b* is 135 for serum, *x* is the volume in μL, and *C* is 0.834 (Ciantar and Caniana 1998). Four readings were recorded for each patient, the volume was calculated for each reading before calculating the average of the four volumes. After that, the GCF periopapers were eluted by performing two cycles of adding 50 μL of eluent buffer (phosphate‐buffered saline (PBS) and Protease Inhibitor Cocktail (PBS‐PIC) (complete ULTRA Tablets, Mini, EDTA‐free, EASYpack catalog number 05892791001) followed by centrifugation at a speed of 11,000 rpm, temperature 4°C, and for 15 min.

**Serum samples**: Four ml of whole blood was collected in a serum separator tube and then centrifuged at 4000 rpm for 5 min to extract the serum and store it at −80°C.

### 2.6 Enzyme‐Linked Immunosorbent Assay (ELISA)

All samples were defrosted before diluting. Saliva samples were spun using Thermo Scientific Fresco 21 Microcentrifuge at 13,000 rpm, 4°C, for 4 min to extract the supernatant, while eluted GCF and serum samples were used as is. Total IgG was assayed using pre‐coated ELISA plates (ThermoFisher; catalog number BMS2091). For the assay, samples were diluted in the kit's assay buffer containing 10 mL assay buffer concentrate 20× (PBS with 1% Tween 20, 10% BSA) and 190 mL distilled water. Saliva was diluted 1:500, eluted GCF 1:100, and serum 1:500,000.

The samples were analyzed in duplicate. The kit's 96‐well plate was precoated with a capture antibody, monoclonal antibody to human total IgG. The detecting antibody was HRP‐conjugate anti‐human total IgG monoclonal antibody. Following three cycles of washes using wash buffer containing 50 mL wash buffer concentrate 20× (PBS with 1% Tween 20) and 950 mL distilled water to remove unbound HRP‐labeled antibody, 100 μL of a room‐temperature substrate solution (tetramethyl‐benzidine) was added to visualize the bound HRP‐labeled antibody The incubation time for each step was 30 min and substrate color development was monitored stopped at 15 min using 100 μL of 1 M Phosphoric acid and absorbance measured at 450 nm (Thermo Scientific Multiskan FC plate reader, catalog number N07710).

The IgG concentration range was calculated by multiplying the IgG concentration (ng/mL) of each saliva, eluted GCF, and serum sample by the sample dilution factor.

### 2.7 Sample Size Calculation

The outcome of interest was the IgG levels in young patients with C/MIP compared to controls. The sample size calculation was based on the means and standard deviations extracted from previous studies, mainly on serum IgG, in a systematic review and meta‐analysis to assess biomarkers in different biological samples (Alamri et al. 2023). To reach a study power of 80%, 62 patients (31 cases and 31 controls) were required. The population mean difference of IgG was μ1 − μ2 = 16.3125 − 20.642 = −4.3295 with standard deviations of 6.9075 and 6.526, respectively and with a significance level (alpha) of 0.05 using a one‐sided two‐sample unequal‐variance t‐test.

### 2.8 Statistical Analysis

Excel was used to input data and check for input errors. ELISA data were organized by subtracting the mean of the no‐sample blanks from all sample absorbances to eliminate background noise and then plotting the standard curve to calculate the sample concentrations in ng/mL. The statistical analysis included three sets of variables: (1) dependent variables, including saliva GCF and serum IgG; (2) demographic covariates, including age, gender, ethnicity, and BMI; and (3) one explanatory variable, diagnosis, including C/MIP and controls. Covariates were considered to reduce potential biases and control their impact on the outcome. SPSS was used to check the normality of data using Kolmogorov Smirnov, which revealed that demographics and IgG data were not normally distributed, unlike clinical scores (BOP, BPE, PPD, and CAL). Therefore, the Mann‐Whitney test was performed for demographics and IgG data was log‐transformed to normalize the distribution before conducting an independent *t*‐test. Pearson correlation coefficient and multiple linear regression were calculated to assess associations between each sample type and other factors. The *p*‐value was < 0.05 for all analyses, and Bonferroni correction was applied for gender and ethnicity subgroup analyses. Python programming language and its libraries: pandas (McKinney 2010), seaborn (Waskom 2021), numpy (Harris et al. 2020), matplotlib (Hunter 2007), and scipy.stats (Virtanen et al. 2020) was used to run statistical tests to plot all the figures.

## 3 Results

### 3.1 Patient Factors

Sixty‐two saliva, 62 GCF, and 30 serum samples were collected from cases with C/MIP and controls. Thirty‐two patients refrained from giving blood samples due to fear of needles given their young age. Patients ranged from 8 to 25 years old, with most females in both groups, Afro/Caribbean ethnicity in cases and Caucasian in controls. Both groups' body mass index (BMI) was normal and had similar BEWE. None of the demographic characteristics showed statistically significant differences between both groups (*p* > 0.05), while the four clinical scores showed a statistically significant increase in C/MIP than controls (*p* < 0.001) (Table 1).

**Table 1:** Demographics of C/MIP and controls.

Variables	C/MIP (*n* = 31)	Controls (*n* = 31)	*p*‐value
**Age**, Median (IQR) (age range)	18 (6) (15–25)	22 (5) (8–25)	0.085
Gender, *n* (%)			0.056
Female	18 (58)	25 (81)	
Male	13 (42)	6 (19)	
Ethnicity, *n* (%)			0.764
Caucasian	6 (19.4)	13 (42)	
Asian	8 (25.8)	11 (35.4)	
Afro/Caribbean	14 (45.1)	3 (9.7)	
Mixed	3 (9.7)	4 (12.9)	
BMI, (kg/cm^2^) Median (IQR)	23 (5.9)	21.9 (4.7)	0.439
GCF vol., (μL) Median (IQR)	0.50 (0.36)	0.40 (0.32)	0.085
BEWE, Median (IQR)	2 (5)	1 (6)	0.133
BOP, μ ± SD	33.45 ± 23.43	15.39 ± 19.55	0.004
Mean BPE, μ ± SD	3.44 ± 0.47	0.93 ± 0.59	< 0.001
Mean PPD, μ ± SD	3.18 ± 0.87	1.91 ± 0.60	< 0.001
Mean CAL, μ ± SD	3.17 ± 0.9	1.75 ± 0.79	< 0.001

Abbreviations: BEWE, cumulative basic erosive wear examination; BMI, body mass index; BOP, full mouth bleeding on probing; BPE, basic periodontal examination; CAL, clinical attachment loss; GCF vol., gingival crevicular fluid volume; PPD, periodontal probing depth.

### 3.2 IgG Data

The range of IgG in C/MIP was 4027–53,576 ng/mL in saliva, 3018–9955 ng/mL in eluted GCF, and 7,288,396–18,286,689 ng/mL in serum, while controls had 2021–36,982 ng/mL in saliva, 928–10,479 ng/mL in eluted GCF, and 7,680,887–14,540,956 ng/mL in serum.

**Figure 1:**
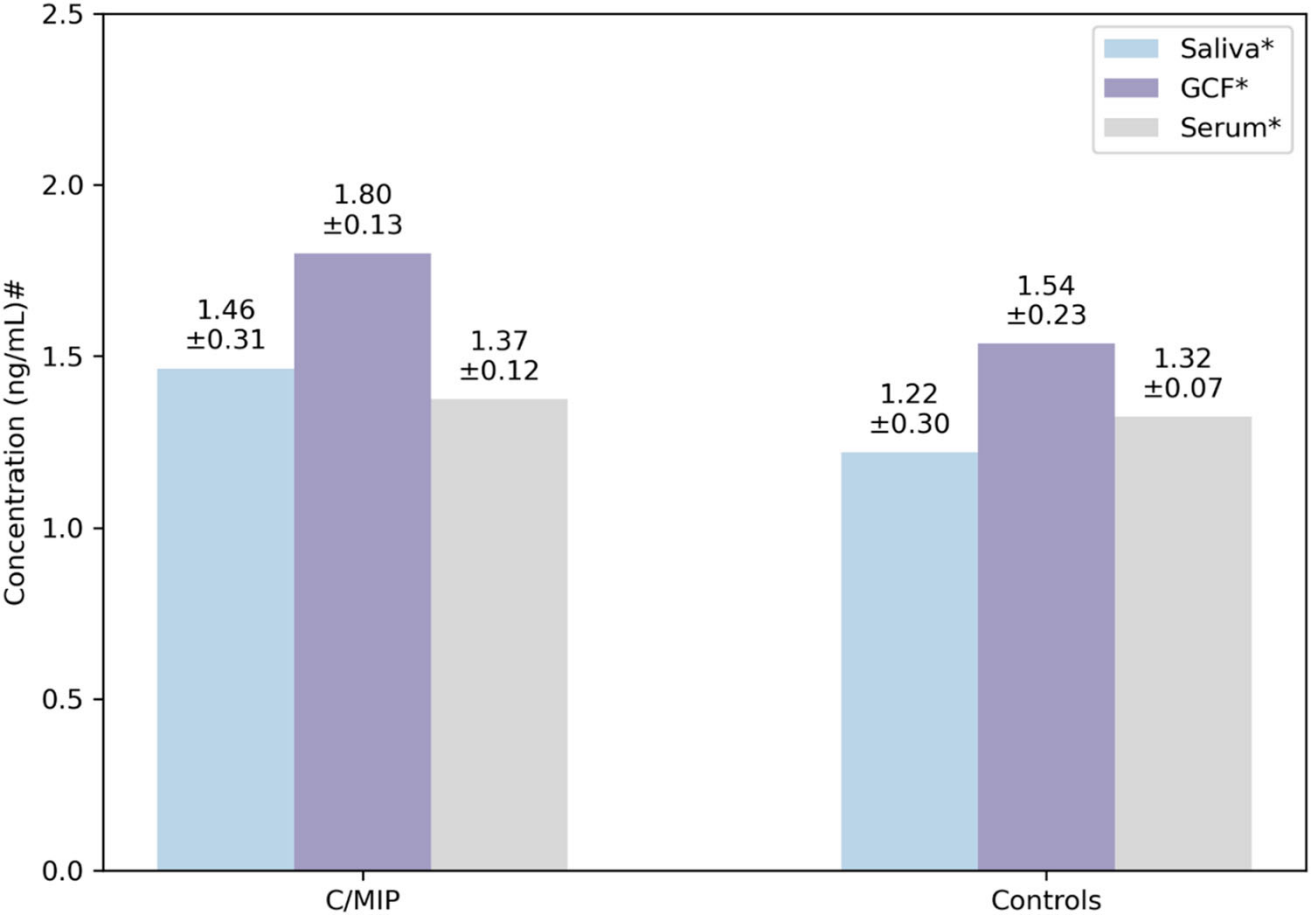
Means and standard deviations of IgG (ng/mL) assayed in diluted samples from C/MIP and controls. *Diluted samples, ^#^log‐transformed values.

The C/MIP group had higher IgG levels in saliva (*p* = 0.003) and GCF (*p* < 0.001) than controls, whereas serum IgG showed no statistically significant differences between C/MIP and controls (*p* = 0.187). Based on the mean values, the IgG in C/MIP was higher in GCF, followed by saliva (Figure 1).

### 3.3 Linear Correlations

Based on Pearson correlation coefficients and its p‐values, C/MIP had higher IgG correlations with saliva (*r* = −0.377, *p* = 0.003) and GCF (*r* = −0.578, *p* < 0.001) than controls; however, the correlation in serum was weak and not statistically significant (*r* = −0.247, *p* = 0.187). Among patient factors, none of the demographics were significantly associated with either saliva or GCF; only gender showed a statistically significant correlation, with males having higher serum IgG than females (*r* = 0.468, *p* = 0.009). The four clinical scores including BOP, BPE, PPD, and CAL displayed significant positive correlations with saliva (*p* < 0.001, < 0.001, 0.010, < 0.001, respectively) and GCF IgG (*p* = 0.039, < 0.001, < 0.001, < 0.001), whereas only PPD (*p* = 0.018) and BOP (*p* = 0.023) were significantly positively correlated with serum IgG (Data not reported in tables).

**Figure 2:**
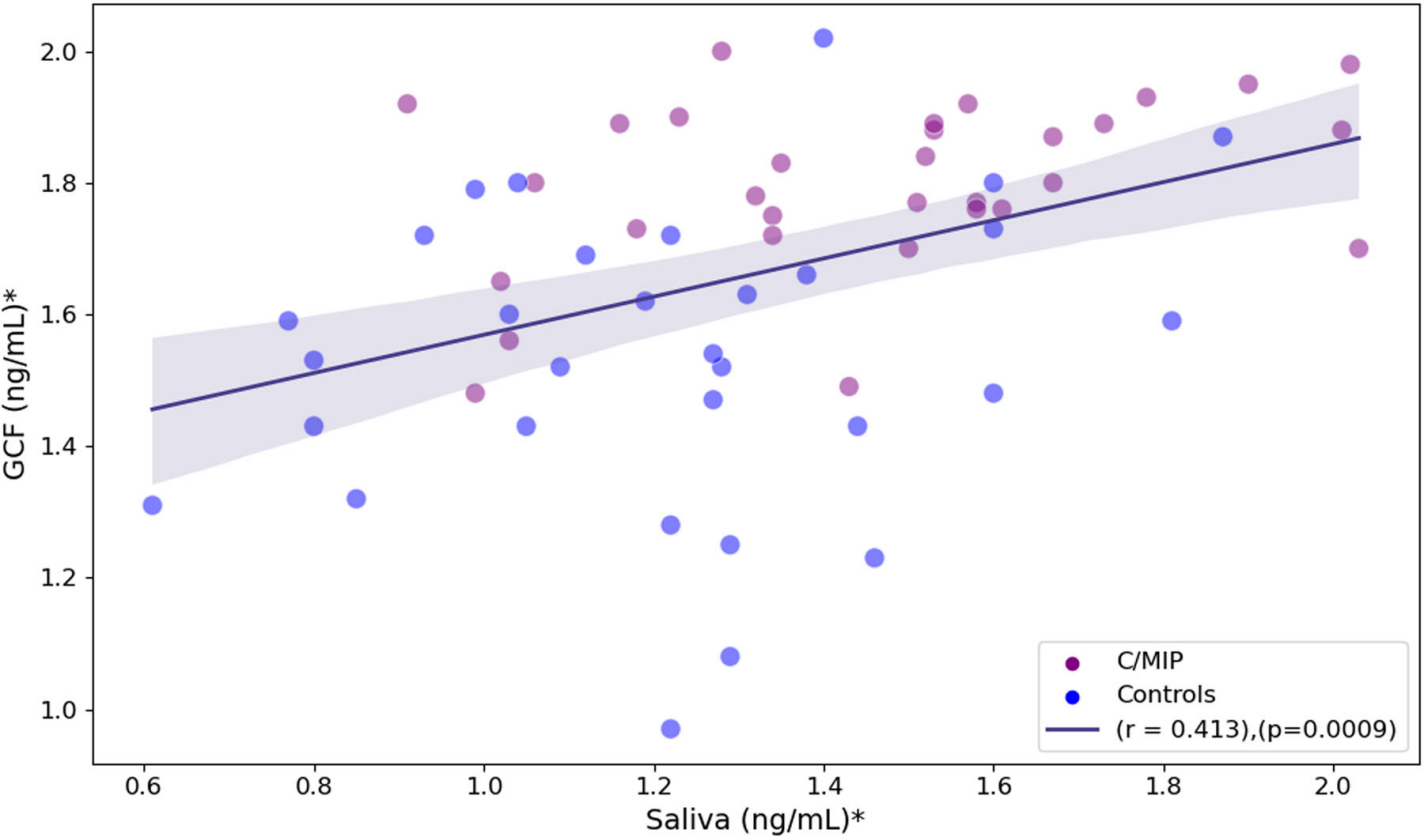
Pearson correlation between saliva and GCF IgG concentrations. *Log‐transformed values.

Among correlations across sample types, a significant positive correlation was observed between saliva IgG and GCF IgG (*r* = 0.413, *p* < 0.001; Figure 2). In contrast, serum showed a slight tendency for a negative correlation with both saliva and GCF (Data not reported in figures). Additional analysis was run for C/MIP to investigate IgG levels and disease severity based on the number of PPD sites exceeding 4 mm. Disease severity was not statistically significantly correlated with saliva IgG (*r* = 0.262, *p* = 0.155), GCF IgG (*r* = 0.120, *p* = 0.520), and serum IgG levels (*r* = ‐0.452, *p* = 0.060) (data not reported in tables).

### 3.4 Multivariate Regression

**Figure 3:**
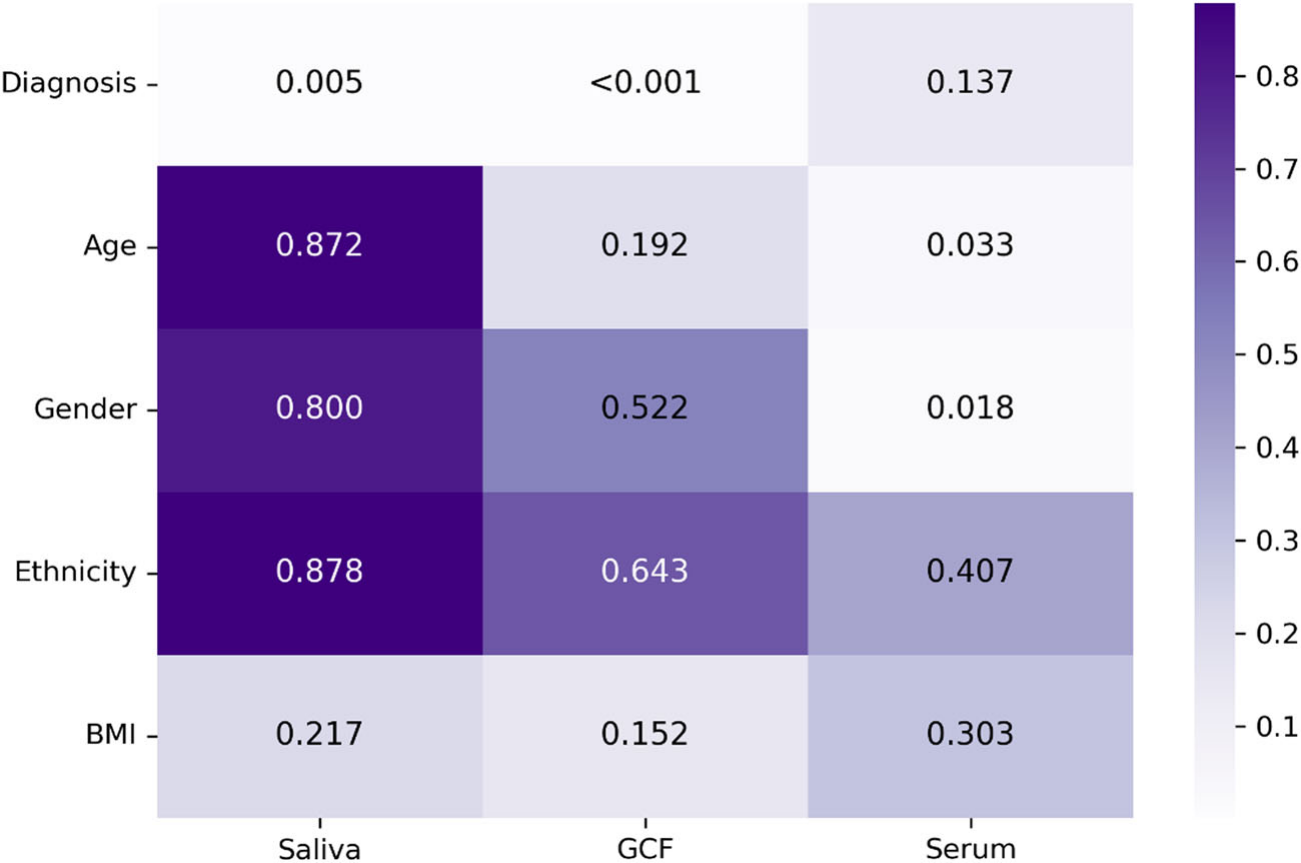
Multivariate regressions between IgG levels and covariates.

Figure 3 represents the *p*‐values (< 0.05) of associations between IgG and diagnosis while controlling for demographic covariates. Three separate analyses were carried out for each sample type. C/MIP had higher IgG levels in saliva (*p* = 0.005) and GCF (*p* < 0.001), unlike serum (*p* = 0.137). Two significant predictors were associated with serum IgG: gender and age: males had higher serum IgG than females (*p* = 0.018), and the concentration of serum IgG increased with age (*p* = 0.033).

Subgroup regression analyses for gender and ethnicity were performed after controlling for demographic covariates. C/MIP males had statistically significantly higher saliva IgG (*p* = 0.018) than control males, whereas CMIP females (*p* = 0.001) and males (*p* = < 0.001) had statistically significantly higher GCF IgG than control females and males. Among ethnicity subgroups, C/MIP Caucasians had statistically significantly higher saliva (*p* = 0.011) and GCF (*p* = 0.003) IgG levels than controls Caucasians. Additionally, Asian C/MIP had higher GCF IgG than controls (*p* = 0.011). None of the subgroups had a significant association with serum IgG (Appendix S1).

## 4 Discussion

To the best of our knowledge, this is the first study to assess IgG levels in three types of samples from young patients with C/MIP aged 25 and younger, filling the gap highlighted in our earlier review of the need to investigate IgG in other samples (Alamri et al. 2023). The current study findings were consistent with the proposed hypothesis since C/MIP had higher IgG levels in saliva and eluted GCF than in controls. GCF IgG was higher in juvenile periodontitis (JP) (former classification of C/MIP) than in controls (Bártová et al. 1995). Serum IgG did not show a statistically significant difference, which aligns with the previous two studies on 13 subjects with early‐onset periodontitis (EOP; the former classification of C/MIP) (Albandar et al. 2002) and 15 with JP (Sandholm and Saxén 1983). Conversely, another study showed that IgG levels were higher in 10 patients with JP than in 9 controls (*p* < 0.02) (Johnson et al. 1980). This contradiction could be due to multiple variations, including laboratory sample processing and patient demographics, for example, in the Johnson et al. study, the age range of controls was 22–42 years, unlike in the other two studies, where they were strictly younger than 25 years. Saliva IgG is known to be elevated in patients with chronic and aggressive periodontitis (Giannobile et al. 2009), and our saliva findings indicate that IgG was elevated in young C/MIP patients. However, these previous studies were statistically underpowered.

### 4.1 C/MIP Pathogenesis

The increase of IgG levels in C/MIP cases compared to controls could be explained as follows. When pathogenic bacteria colonize the periodontal sulcus, they secrete toxins to damage periodontal tissue (Abdulkareem et al. 2023; Leonardo et al. 2004). The secretion of toxins triggers the host immune system to react and respond by recruiting the first line of white blood cells, including neutrophils and macrophages (Dahiya et al. 2013; Hajishengallis et al. 2020; Kornman et al. 1997). The adaptive immune system is activated since the inflammation is chronic and aggressive in C/MIP (Cekici et al. 2014). B‐cells, responsible for IgG synthesis, are among the cells attracted to the site of inflammation where certain bacteria trigger the production of specific antibodies (Figueredo et al. 2019). IgG is fundamental in neutralizing bacterial toxins and marking them for destruction (Burton 1990). As a result of this inflammation, GCF flow increases, and both saliva and GCF contain high levels of antibodies, including IgG (Giannobile et al. 2009; Subbarao et al. 2019), consistent with our findings. It is worth mentioning that the host system reaction can be exaggerated, leading to excessive inflammatory responses (Ricklin et al. 2016). With the increased production of IgG, more bacteria are opsonised and phagocytosed by neutrophils and macrophages (Peeran and Ramalingam 2021b). Reactive oxygen species (ROS) and proteases are secreted excessively during phagocytosis, leading to periodontium protein breakdown and tissue destruction (Dupré‐Crochet et al. 2013; Sies 1997). Moreover, overproduction of IgG could also contribute to tissue destruction (Yanaba et al. 2008). Conversely, healthy periodontium tends to have lower numbers of B‐cells and neutrophils, resulting in a lower IgG output (Hajishengallis et al. 2020). Furthermore, autoantibodies have been used as diagnostic biomarkers (Yanaba et al. 2008). In this context, IgG production increases when the disease is initiated or progresses, and it decreases in recovery and health.

Although this study has focused on total IgG, the subclass IgG2, being specific for the carbohydrate antigens of periodontal pathogens such as *Actinobacillus actinomycetemcomitans* and *Porphyromonas gingivalis*, was associated with aggressive periodontitis (Albandar et al. 2002; Lu et al. 1994; Schenkein et al. 2007). In fact, the elevation of IgG2, especially in localized aggressive periodontitis, suggests that a specific response against these pathogens is taking place (Diehl et al. 2003), thus suggesting its contribution to the pool of total IgG in such cases.

### 4.2 IgG Concentrations

The current study identified a positive correlation between saliva IgG and GCF IgG concentration. This corroborates the findings of Stefanović et al. who investigated IgG levels in saliva and GCF from older patients with advanced periodontitis and found IgG to be equally increasing in both fluids as inflammation increases (Stefanović et al. 2006). We also observed that GCF had a higher concentration of IgG than saliva, which is in line with the fact that there are high levels of IgG in GCF (Peeran and Ramalingam 2021a). Moreover, the IgG in GCF is derived from the body's circulation and secreted locally in the gingival sulcus/pocket (Peeran and Ramalingam 2021b; Tew et al. 1985; Tollefsen and Saltvedt 1980), which could eventually equal or exceed the IgG concentration in serum (Holmberg and Killander 1971; Tew et al. 1985). IgG ranges of concentration differ depending on the sample type: in controls, it is estimated to be 20–30 µg/mL in saliva (Engström et al. 1996) and 7000‐16,000 µg/mL in serum (Dati et al. 1996), while in GCF, it varies from site to site in the same person and with age (Tew et al. 1985). Based on our previous systematic review focusing on young C/MIP (Alamri et al. 2023), only two studies reported serum IgG ranges in C/MIP of 8500–17,800 µg/mL (Sandholm and Saxén 1983) and 7900–18,800 µg/mL (Sjödin et al. 1995) while no study assessed IgG in saliva, and the one study on GCF did not report the range (Alamri et al. 2023). Despite the low IgG concentration in saliva compared to serum, IgG in saliva was considered a potential salivary biomarker in periodontal diseases (Proctor 2016). Compared to these values, our saliva IgG range in controls was slightly higher, unlike serum IgG, which was within the range for controls and C/MIP. Regarding the GCF range, no previous paper reported IgG ranges in GCF, so this could not be compared.

The weak negative correlation between serum IgG and saliva and GCF IgG in both groups did not reach the statistical threshold. No previous study assessed IgG correlations between different samples from young C/MIP. However, a study on 10 patients with other forms of periodontitis reported a significant correlation between serum and GCF IgG, with GCF showing a higher concentration than serum, yet they did not specify the correlation direction (Tollefsen and Saltvedt 1980). This discrepancy could be due to multiple reasons, including differences in periodontal conditions, age, and number of included participants.

### 4.3 Patient Factors

In the overall regression model, none of the patient factors was significantly associated with saliva or GCF. Nonetheless, gender and age were associated with serum IgG. Males showed higher serum IgG levels than females. Also, the IgG concentration seems to be higher in older ages, possibly due to the progression of periodontitis with age. Both findings cannot be compared to previous studies as those studies did not correlate serum IgG in young C/MIP with age and gender. However, in the subgroup regression by gender, C/MIP males had higher saliva IgG than control males and both genders in the C/MIP group had higher GCF IgG than both genders in the control group, suggesting the potential impact of C/MIP on saliva IgG in males and on GCF IgG levels in both genders. In addition, ethnicity subgroup analysis revealed that C/MIP Caucasians had elevated saliva and GCF IgG than control Caucasians, while surprisingly, Afro‐Caribbean showed no significant associations despite their numbers exceeding Caucasians. Furthermore, Asian C/MIP had higher GCF IgG than Asian controls. These findings cannot be compared to previous studies due to the absence of comparable studies on gender and ethnicity IgG in C/MIP cases. Still, there is a lack of data regarding this aspect, especially with the host immune response involved (Tavakoli et al. 2022).

### 4.4 Clinical Implications

Saliva and GCF sample collection is easy, noninvasive, and provides valuable insight into the underlying host response (Barros et al. 2016; Jaedicke et al. 2016). The assay of IgG in saliva and GCF can be considered diagnostic in patients with C/MIP, especially GCF, since it is mainly localized to the site of infection rather than saliva or serum, which are more involved with salivary gland and systemic conditions (Poorsattar Bejeh‐Mir et al. 2014).

### 4.5 Strengths and Limitations

This study focused on total IgG levels in young C/MIP patients, thus filling this gap in the literature. IgG was investigated in three samples using ELISA specialized kits to ensure sensitive detection of IgG and enhance the findings' robustness. The study provided estimated ranges for IgG in eluted GCF in C/MIP and controls. Nevertheless, some limitations were encountered: (1) the relatively small sample size, (2) the analysis of total IgG only, (3) less serum samples compared to saliva and GCF, (4) although none of the patients exceeded 10 min for saliva samples collection, the exact time for each patient was not recorded (5) samples were collected from patients at different times based on their availability, and (6) The inclusion of few cases that had few sites involved more than two teeth.

## 5 Conclusion

Our previous work revealed a statistically significant increase of serum IgG in C/MIP compared to controls (Alamri et al. 2023) and revealed the paucity of studies on total IgG in other samples, including saliva and GCF. It led to conducting this study, which concluded that young patients with C/MIP had elevated IgG levels in saliva and GCF, compared to controls. Both saliva and GCF samples exhibited positive linear association: IgG increases or decreases in both. Consequently, IgG could be a potential biomarker in C/MIP; however, these findings cannot be generalized due to the small sample size. Indeed, there is a lack of recent studies on IgG in C/MIP, so future studies with larger sample sizes are needed to investigate IgG extensively and validate its sensitivity and specificity as a diagnostic biomarker of C/MIP.

## Author Contributions

L.N., G.P., and M.M.A. contributed to the study design. M.M.A. and L.N. contributed to the investigation. M.M.A. conducted the laboratory analysis of the samples, extracted data, performed data analysis, and drafted the manuscript. M.M.A., G.P., and L.N. reviewed and edited the manuscript. All authors read and approved the manuscript and agreed to the submission of the manuscript to the journal.

## Ethics Statement

The Biobank received ethics approval from the East of England‐Cambridge East Research Ethics Committee (reference 20/EE/0241).

## Consent

Each participant provided written consent to participate in the Biobank, and the Biobank Management Committee (Biobank reference REF018) granted specific approval to release data and samples for this study.

## Conflicts of Interest

All authors declare no conflicts of interest.

## Supporting information

Supporting information.

## Data Availability

The data supporting this study's findings are available on request from the corresponding author.
