# Supplementary material for: IgG In Saliva, GCF, and Serum in Young Patients With Grade C Molar Incisor Pattern Periodontitis
Source: Clin Exp Dent Res. 2025 Mar 30;11(1):e70117. doi: 10.1002/cre2.70117 (PMC11955182; doi:10.1002/cre2.70117)
Supplement: Supplementary file 1 — Supporting information. [file CRE2-11-e70117-s001.docx]

**Table 2. Correlations between gender and ethnicity and IgG levels in saliva, GCF, and serum.**

|  | **Subgroups** | **Saliva IgG**  n(C/MIP, Controls)  r (p-value) | **GCF IgG**  n(C/MIP, Controls)  r (p-value) | **Serum IgG**  n(C/MIP, Controls)  r (p-value) |
| --- | --- | --- | --- | --- |
| **Gender^*^** | Female | n=(18, 25)  -0.158 (0.052) | n=(18, 25)  -0.216 (0.001) | n=(10, 10)  -0.019 (0.350) |
|  | Male | (n=13, 6)  -0.397 (0.018) | (n=13, 6)  -0.418 (<0.001) | n=(8, 2)  -0.153 (0.064) |
| **Ethnicity^**^** | Caucasian | n=(6, 13)  -0.319 (0.011) | n=(6, 13)  -0.387 (0.003) | n=(4, 5)  0.087 (0.168) |
|  | Asian | n=(8, 11)  -0.188 (0.062) | n=(8, 11)  -0.210 (0.011) | n=(6, 6)  0.029 (0.203) |
|  | Afro/Caribbean | n=(14, 3)  -0.126 (0.171) | n=(14, 3)  -0.172 (0.047) | n=(8, 1)  -0.086 (0.013) |
|  | Mixed | n=(3, 4)  -0.672 (0.127) | n=(3, 4)  -0.153 (0.138) | - |

**^*^**Bonferroni p-value was applied at <0.025, **^**^** Bonferroni p-value was applied at <0.0125
